# New Literature

**DOI:** 10.1155/S146392467900078X

**Published:** 1979


### New Literature

### Plasma emission spectroscopy

A paper is available which describes atomic spectroscopy using the SMI Spectrospan III plasma emission spectrometer. It outlines each of the instrumentation modules – DC argon plasma source, échelle grating spectrometer, optical cassettes, and use of a controlling microprocessor. Applications are described in the fields of environmental analysis of toxins, biological materials, and natural and effluent waters; analysis of impurities and major element contents of materials used for microcircuitry construction; trace element determinations in stainless steels; wear-metal detection in heavy machinery lubricating oils for engine assessment and analysis of trace metal content of gelatin emulsion used in the photographic industry.

*Techmation Ltd., 58 Edgeware Way, Edgeware, Middx HA8 8JP, UK.*

### Spectrodensitometer

The SD 3000 Spectrodensitometer for the qualitative analysis of thin layer chromatograms is described in an 8-page brochure recently published by the Schoeffel Instrument Division of Kratos. The SD 3000 is a thin layer chromatogram scanner capable of making absorbance and fluorescence measurements of spotted thin layer separations.

*Kratos Inc., Barton Dock Road, Urmston, Manchester M31 2LD, UK.*

### Switches

Most switch contacts are open to the environment, but high pressures, water, corrosive and volatile fluids can all cause electrical equipment to fail. A new leaflet from Techmation describes the Giannini range of Envir-Switches, the contacts of which are enclosed within a welded chamber and are operated externally by magnetic action. The leaflet describes the available switch materials and such configurations as push button switches, rotary switches, plungers, key-lock switches and limit switches.

*Techmation Ltd., 58 Edgeware Way, Edgeware, Middx, UK.*

### Spectroscopy

Issue 15 of Rank Hilger News contains information on the new computing electronics available with their optical emission spectrometers, Polyvac Systems E 960 and E1000 embodying a microprocessor based control and data processing system. A new multichannel liquid analyser, the Chemispek, is also featured.

*Hilger, Westwood, Margate, Kent CT9 4JL, UK.*

### Radioassay and chromatography supplies

Two new brochures are now available from Packard describing their range of equipment for radioassay and chromatography. 'Chemicals and supplies for radioassay' is a 24-page booklet giving details of scintillation consumables, including scintillation cocktails, reagents, vials and reference materials for liquid scintillation, and automatic gamma counting techniques.

The second brochure, Chromatography supplies is a fully illustrated catalogue of ancillary chromatography equipment ranging from a variety of absorbants to solvent dispensers.

*Packard Instruments Ltd., 13-17 Church Road, Caversham, Berks, UK.*

### UKAEA publications

The United Kingdom Atomic Energy Authority, Harwell have published a number of papers which may be of interest. A Harwell 6000 series microprocessing system by G.I. Phillips describes the design and use of a group of Harwell 6000 series microprocessing modules based on an Intel 8085 processor. A number of systems which have been developed using these modules are also described.

A paper by K.D. Boness, 'A transducer position controller for automatic scanning describes a simple CAMAC–6000 series interface', which allows an automatic data processing program to control mechanical movement of a scanning transducer. Evaluation of high resolution gamma spectrometry for in-line determination of uranium and plutonium in distribution coefficient measurements by D. Scargill of the Chemical Technology Division, A.E.R.E., Harwell is also available.

*These publications and the UKAEA List of publications available to the public (price £2.00) can be obtained form HM Stationery Office.*

### Liquid chromatography application

The Finnigan Corporation have published an application report entitled The determination of herbicide photolysis products by LC/MS, by R.F. Skinner and Q. Thomas of Finnigan, and J.Giles and D.G. Crosby of the University of California at Davies. The paper describes the analysis by liquid chromatography and mass spectrometry (LC/MS), or oryzalin, a pre-emergence herbicide, and its photodegradation products.

*Finnigan's Publications Department, 845 West Maude Avenue, Sunnyvale, California 94086, USA.*

### Automation of COD analysis

A paper is available from Techmation which describes the automatic measurement of chemical oxygen demand by Johnson COD meter in the sewage systems of a Swedish pulp mill.

*Techmation Ltd., 58 Edgeware Way, Edgeware, Middx HA8 8JP, UK.*

### Thermal analysis

Stanton Redcroft have published a booklet giving full details and specifications of their STA simultaneous thermal analysis system.

*Stanton Redcroft, Copper Mill Lane, London SW17 OBN, UK.*

### Electrophoresis

Bio-Rad Laboratories have introduced a package of literature describing the applications of their model 1415 multipurpose electrophoresis cell. The literature looks in depth at iso-electrofocusing techniques describing new methods for gel drying and fraction locating in preparative electrofocusing and their thin layer acrylamide casting stand for analytical electrofocusing. The utilisation of a reversible cooling stage and the importance of a cooling coil for the collection condensation within the electrophoresis cell is explained.

*Bio-Rad Laboratories Ltd., Caxton Way, Holywell Industrial Estate, Watford, Herts WD1 8RP, UK.*

### Chromatography data processor

Dyson instruments have made available a booklet on the Shimadzu Chromatopac C–R1A recording data processor for chromatography. The booklet gives full details of the function, specification and accessories for this instrument.

*Dyson Instruments, 4 Frosterley Close, Newton Hall, Durham.*

### NMR catalogue

Kontes have issued a new NMR catalogue. The 12-page, illustrated booklet includes sections on sample preparation, sample handling, sample tubes, microcell assemblies, EPR-ESR, special tubes and accessories.

*Kontes, PO Box 729, Vineland, New Jersey 08360, USA.*

Meeting Reports *(Continued from page 287)*
determination of viscosity by flow injection analysis.

The final lecture session of the conference was chaired by Dr. Betteridge and started with a paper delivered by Dr. K. Brunt on the design of an amperometric flow detector based on a rotating disc electrode. This was followed by a paper from Dr. M. Trojanowicz of the University of Warsaw who described a system for the continuous determination of sulphate using a differential flow system. The final paper, by Dr. Alan Townshend from Birmingham, described an extremely novel system enabling chemiluminescent detectors to be applied to flow injection.

Dr. Betteridge then gave some light-hearted suggestions for solving the problem of nomenclature, after which Dr. van der Linden presented flowers to Ms. Ellen Abeling and Mrs. Tilly Sijpesteyn who had been responsible for running the conference office and who had provided much invaluable help.

Finally, the conference was formally closed and everyone came away having learned a great deal about the state of the art of flow analysis, and having spent a very pleasant week in one of the most beautiful cities of Europe.

Proceedings of the conference will appear in a special edition of Analytica Chemica Acta to be published shortly.

**Tim Sly**

### Book Reviews

### Laboratory Engineering and Manipulations: 3rd Edition

*Edited by E.S. Perry and A. Weinsberger. John Wiley & Sons, Chichester, 1979, pp 531 + xi, £26.80*
*ISBN 0471-032-751*

In this, the third edition, the work has been reorganised and most parts have been thoroughly revised and rewritten. The chapter on 'Operations with Gases' has been deleted as little progress has been made in this technique since the previous edition. The work is now composed of eight chapters, each contributed by an expert or group of experts and covering a fundamental aspect of laboratory engineering, and each is subdivided into subject sections.

As an example the first chapter 'Selection of Materials for the Construction of Equipment' starts with a short introduction describing mechanical, electrical and thermal properties of materials; continues with a fairly detailed account of the corrosion of metals and the ways in which protection against corrosion may be achieved; briefly looks at non-metals, and concludes with a section dealing with the practical evaluation of chemical resistance. Included are tables referring to the general chemical resistance of common metals and general properties of a number of non-metals.

Other chapters are titled 'Laboratory Heat Transfer', which covers the whole field of temperature measurement and control, heating, cooling and insulation; 'Grinding, Blending, Screening and Classifying', 'Pumps and Flow Measurement'; 'Glove Box Technique'; 'Mixing'; 'Vacuum Technique'; and 'Solvent Removal, Evaporation, and Drying'. In each of these major aspect chapters a mathematical description is given where it appears necessary, types of equipment ranging from simple laboratory scale to small industrial scale are described and illustrated, and an extensive list of references on 'Solvent Removal' deals with the subject in a too mathematical way with little information on available equipment. The index of twenty-three pages would appear to be perfectly adequate.

The book is well produced with numerous figures, tables and illustrations of a consistent quality. It is doubtful whether it would find much use in the small analytical laboratory except as background reading, but in a larger organisation its depth of detail on such a wide range of subjects would make it a well used reference book.

*T.G. Alliston*

### Product News

### Desk-top computer

Hewlett-Packard have introduced the latest addition to their range of modular desk-top computers, the System 45B. System 45B features a standard memory of 56266 bytes, which is expandable up to a maximum of 448,906 bytes. The operating system is held in its own room so all this memory is available to the user. It also has built-in mass storage with two tape cartridge drives (one optional).

Other features include a 31 cm CRT with a standard alphanumeric mode and a graphics option. In the alphanumeric mode, the CRT can display up to 1920 characters, and the graphics option can be used to generate and display data plots, two and three dimentional drawings, charts, etc. A copy of the display can be made by the internal printer/plotter. A range of standard peripherals can also be linked to the system. System 45B has a typewriter-style keyboard with alphanumeric control, editing keys, and 32 user-definable, special function keys, and uses enhanced ANSI BASIC language. In addition to the standard keyboard, optional keyboards with special characters for German, French, Spanish and Scandanavian languages are available. The Hewlett-Packard library of software packs enables the system to be quickly tailored to application requirements such as engineering design, business management, statistical analysis, financial planning and instrument control.

*Hewlett-Packard Ltd., King Street Lane, Winnersh, Wokingham, Berks RG11 5AR, UK.*

### Karl Fischer titrator

The Photovolt Aquatest Mk IV Automatic Karl Fischer Titrator is available in the UK through Auriema. It provides a direct digital readout of the water level in liquids and solids within the range 10µg to 10mg. A microprocessor control provides a simple moisture determination. Water is titrated by electrolytic generation of Karl Fischer reagent. The inclusion of microprocessor facilities in the instrument eliminates the need for standardisation, the need to add or measure reagents and correction due to interfacing reactions. It also eliminates lengthy calculations.

Other features included are the facility to provide a programmable extraction time, automatic correction for interfering reactions, and automatic blank correction when extracting. Analyses can be carried out in the presence of anti-oxidants and compensation is made for small reactions due to carbonyl compounds.

*Auriema Ltd., 442 Bath Road, Slough SL1 6BB.*

### $CO_2$ analyser

Vickers Medical have developed a $CO_2$ analyser for use in operating theatres and intensive care units. The CD-300 analyser measures end-tidal $CO_2$ concen-

